# Environmental impacts of dietary shifts in India: A modelling study using nationally-representative data

**DOI:** 10.1016/j.envint.2019.02.004

**Published:** 2019-05

**Authors:** L. Aleksandrowicz, R. Green, E.J.M. Joy, F. Harris, J. Hillier, S.H. Vetter, P. Smith, B. Kulkarni, A.D. Dangour, A. Haines

**Affiliations:** a Dept. of Population Health, London School of Hygiene & Tropical Medicine, UK; b Leverhulme Centre for Integrative Research on Agriculture & Health, UK; c Royal (Dick) School of Veterinary Studies, University of Edinburgh, UK; d Institute of Biological and Environmental Sciences, University of Aberdeen, UK; e Clinical Division, National Institute of Nutrition, India; f Dept. of Public Health, Environments and Society, London School of Hygiene & Tropical Medicine, UK

**Keywords:** India, Dietary intake, Sustainable diets, Dietary guidelines, Greenhouse gas emissions, Land use, Water use

## Abstract

Food production is a major driver of environmental change, and unhealthy diets are the leading cause of global disease burden. In high-income countries (HICs), modelling studies suggest that adoption of healthy diets could improve population health and reduce environmental footprints associated with food production. We assessed whether such benefits from dietary change could occur in India, where under-nutrition and overweight and obesity are simultaneously prevalent.

We calculated the potential changes in greenhouse gas (GHG) emissions, blue and green water footprints (WFs), and land use (LU), that would result from shifting current national food consumption patterns in India to healthy diets (meeting dietary guidelines) and to “affluent diets” (those consumed by the wealthiest quartile of households, which may represent future purchasing power and nutritional trajectories). Dietary data were derived from the 2011–12 nationally-representative household expenditure survey, and we assessed dietary scenarios nationally and across six Indian sub-regions, by rural or urban location, and for those consuming above or below recommended dietary energy intakes. We modelled the changes in consumption of 34 food groups necessary to meet Indian dietary guidelines, as well as an affluent diet representative of those in the highest wealth quartile. These changes were combined with food-specific data on GHG emissions, calculated using the Cool Farm Tool, and WF and LU adapted from the Water Footprint Network and Food and Agriculture Organization, respectively.

Shifting to healthy guidelines nationally required a minor increase in dietary energy (3%), with larger increases in fruit (18%) and vegetable (72%) intake, though baseline proportion of dietary energy from fat and protein was adequate and did not change significantly. Meeting healthy guidelines slightly increased environmental footprints by about 3–5% across GHG emissions, blue and green WFs, and LU. However, these national averages masked substantial variation within sub-populations. For example, shifting to healthy diets among those with dietary energy intake below recommended guidelines would result in increases of 28% in GHG emissions, 18 and 34% in blue and green WFs, respectively, and 41% in LU. Decreased environmental impacts were seen among those who currently consume above recommended dietary energy (−6 to −16% across footprints). Adoption of affluent diets by the whole population would result in increases of 19–36% across the environmental indicators. Specific food groups contributing to these shifts varied by scenario. Environmental impacts also varied markedly between six major Indian sub-regions.

In India, where undernutrition is prevalent, widespread adoption of healthy diets may lead to small increases in the environmental footprints of the food system relative to the status quo, although much larger increases would occur if there was widespread adoption of diets currently consumed by the wealthiest quartile of the population. To achieve lower diet-related disease burdens and reduced environmental footprints of the food system, greater efficiency of food production and reductions in food waste are likely to be required alongside promotion of healthy diets.

## Highlights

• Uptake of healthy diets in India results in small increases in GHG emissions, water and land use.
• However, national averages mask substantial variation within sub-populations.
• Footprints would increase among those consuming below dietary energy guidelines.
• Conversely, footprints would decrease for those consuming above dietary energy guidelines.
• Uptake of affluent diets would result in increases of 19–36% across environmental footprints.

## 1 Introduction

Food production contributes globally to 19–29% of greenhouse gas (GHG) emissions, 70% of freshwater withdrawals, and uses one-third of ice-free land (Smith et al., 2014; Whitmee et al., 2015; Vermeulen et al., 2012). Food systems face an unprecedented challenge of providing an estimated 60% more food by 2050 to feed a growing and more prosperous population, while food production will likely face increased pressures from climatic and environmental change (Food and Agriculture Organization, 2016a; Myers et al., 2017). Current diets in high-income countries (HICs) contain excess dietary energy and high intakes of animal-based foods, resulting in high per capita environmental footprints (Pradhan et al., 2013; Tilman and Clark, 2014). A growing body of evidence has highlighted the mitigation potential of shifting current HIC diets to those which are healthier and reduce environmental impacts (Aleksandrowicz et al., 2016; Springmann et al., 2016; Green et al., 2015). A variety of more environmentally sustainable dietary patterns have been proposed, with possible reductions in environmental footprints of 30–50% for vegetarian diets (Aleksandrowicz et al., 2016). Achieving widespread uptake of these diets may be challenging, though modest environmental benefits could also be achieved by shifting to national dietary guidelines, which are currently widely supported, and potentially easier to adopt. However, little is known about the impacts of such options in low- and middle-income countries (LMICs) (Aleksandrowicz et al., 2016; Global Panel on Agriculture and Food Systems for Nutrition, 2016a).

Globally, around 45% of countries have significant levels of both under-nutrition and overweight/obesity; approximately 2 billion individuals are overweight or obese, and 800 million have inadequate dietary energy intake (International Food Policy Research Institute, 2016). In this context, increased adoption of healthy diets is critical to reducing all forms of malnutrition, though the impact of such dietary changes on various environmental pressures is uncertain. For example, high-income households may benefit from reducing overall dietary energy intake and replacing at least some consumption of animal-based foods with plant-based foods. In contrast, an increase in diet-related environmental footprints may be necessary for those households aiming to reach adequate dietary energy and diversity. Understanding these dynamics is important to guide policies that will deliver healthy diets and improved nutrition for all individuals, within climate and other planetary boundaries (Food and Agriculture Organization, 2016a; Steffen et al., 2015).

India is home to almost one-fifth of the global population, and has high rates of undernutrition (including one-third of the world's cases of child stunting) coinciding with growing rates of obesity and non-communicable diseases (NCDs) (International Food and Policy Research Institute, 2017a; Registrar General of India and The Centre for Global Health Research, 2015; Shankar et al., 2017). The country also faces critical environmental pressures on its ability to produce food. Despite its large share of the global population, it covers only 2.4% of the world's land (Department of Agriculture CFW, 2016), and agricultural irrigation accounts for 90% of freshwater use despite depleting groundwater reserves in some regions (Food and Agriculture Organization, 2016b; Rodell et al., 2018). Although per capita GHG emissions are relatively low, India is the 4th highest contributor to global GHG emissions, behind China, the US, and the EU (World Resources Institute, 2017), and has committed to reducing emissions under the Paris Climate Agreement (Shrivastava, 2016). Indian diets are transitioning away from staple foods, such as pulses and coarse cereals, to vegetable- and animal-based fats, and energy-dense, highly processed foods (Gaiha et al., 2013; Baker and Friel, 2014; Popkin, 2002), though dietary energy from cereals still remains high (Deaton and Drèze, 2009). As incomes continue to rise, diets are projected to both diversify nutritionally and include excess dietary energy, particularly from oils, meat, dairy, and sugar (Kearney, 2010; Muhammad et al., 2017). Globally, these changes may increase the number of obese individuals from 1.33 billion in 2005 to 3.28 billion by 2030, with Asia leading in the transition from dietary energy insufficiency to excess (Global Panel on Agriculture and Food Systems for Nutrition, 2016b). Economic growth alone will not necessarily improve nutrition (Global Panel on Agriculture and Food Systems for Nutrition, 2016b), and projected dietary changes may also further compound existing environmental pressures.

Recent work has shown that the much-needed shifts to healthy diets in selected Indian regions could partially buffer water-related pressures facing agricultural production, and decrease GHG emissions (Milner et al., 2017), and a national study also concluded that heathy dietary shifts could reduce GHG emissions (Rao et al., 2018). Here, we extend this work by combining, for the first time, nationally-representative dietary data with food-specific GHG emissions, water footprints (WFs), and land use (LU), to assess multiple environmental indicators. We explore two scenarios – a shift to healthy diets, and a shift to “affluent” diets, a perspective that has not previously been studied – to assess the environmental opportunities and challenges of food systems to meet dietary needs in India.

## 2 Methods

### 2.1 Data

Dietary data were derived from the 68th round of the Indian National Sample Survey (NSS), a nationally-representative household consumer expenditure survey conducted in 2011–12 (n = 101,651 households) (National Sample Survey Office, 2014). The questionnaire records the quantity and value of approximately 140 food, meal and beverage items purchased by the household within the last month, among other consumer goods, and we used the quantity of food purchased and produced for own consumption as a proxy for intake. We used the improved “type 2” format of the survey which used 7-day recall for meats, eggs, oils, fruits and vegetables, and 30-day recall for cereals, pulses and sugar. This survey is the only nationally-representative source of quantitative dietary data in India (Aleksandrowicz et al., 2017).

Household-level data on quantity of food purchased was divided out among household members to approximate individual-level intakes, using Indian energy requirement consumption units based on age and sex, as provided in the NSS documentation (National Sample Survey Office, 2014) (the survey included household members of all ages). We adjusted household intake for meals received by members (school meals, payment for labour, etc.), and/or provided to non-household members (further details in Supplementary file 1). These are recorded separately from the food expenditure and would otherwise skew the amount of food available for household consumption from the recorded expenditure; for context, approximately 23% of households received a net positive amount of meals, while 38% provided more meals than received. We calculated dietary energy, protein and fat intake using nutritional composition data provided by NSS documentation for each of 134 food items, and aggregated the intake of these items into 34 food groups based on nutritional content similarity (details of groupings are provided in the Supplementary Table 1). Individuals consuming below 200 or above 5000 kcal/day were excluded (n = 1829), and our final sample of individuals was 462,901. We additionally adjusted intake of the 34 food groups to approximate food group intake from meals eaten out of home (on average, 18% of households' dietary energy; additional details in Supplementary file 1). We used household sample weights in our tabulation of baseline intake of the 34 food groups. We then linked each food group to estimates of GHG emissions, blue and green WFs, and LU associated with the production of food items.

We used existing data on GHG emissions (kg CO_2_-eq/kg food product) that had been derived for the food groups used in this analysis (Green et al., 2018). The values are based on emissions associated with the agricultural production stage of major crops and livestock products, estimated with a derivative of the Cool Farm Tool (CFT) (Hillier et al., 2011; Vetter et al., 2017), using Indian farm-level activity data obtained from the Directorate of Economics and Statistics of the Government of India (http://eands.dacnet.nic.in). The set of empirical models making up CFT use inputs on soil, climate, and farm management, including fertiliser, pesticide and herbicide use, residue management, machinery, and energy use. Emissions from rice production were calculated using the approach of Yan et al. (2005). National-level emission averages were used for food items. CFT was used to derive emissions directly for 22 out of our 34 food groups. For groups that could not be assessed as above, production-stage emissions were derived from the literature, or a CFT-derived proxy was allocated. Production stage emissions were then combined with post-production stage emissions, also based on review of the literature (Green et al., 2018). Where two or more items were aggregated within a food group (i.e., other pulses, other cereals, ruminant meats, etc.), footprints were weighted by the quantity of the individual items consumed. Further details of these data have been published (Green et al., 2018; Vetter et al., 2017).

Data on India-specific WFs (L/kg food product) were used from a previous study that derived footprints for the same food groups and items used in this analysis. The existing values were adapted from a database made publicly available by the Water Footprint Network (WFN) (Mekonnen and Hoekstra, 2011; Mekonnen and Hoekstra, 2012) (http://waterfootprint.org/en/resources/water-footprint-statistics/). Individual product footprints from the WFN data were matched to food groups based on author judgement, and the total footprint of a food group was weighted by the quantity of consumption of individual items within the group. To account for geographical differences in WF values throughout India, we used national values that had weighted average state-level values by land area (see Harris et al. (2017), for description of methods). We assessed both blue (ground and surface) and green (rainfall) WFs.

Land use (m^2^/kg food product) for crops within our food groups was derived directly from FAO yield data for India for the year 2014 (Food and Agriculture Organization of the United Nations, 2017). For livestock products, FAOSTAT publish data on yields per head of livestock but not yields per unit area of land. Thus, yield data for livestock products were calculated on the basis of livestock feed requirements (Mekonnen and Hoekstra, 2012), yields of feed crops and fodder (Food and Agriculture Organization of the United Nations, 2017; Shankar and Gupta, 1992), and feed conversion efficiencies. Nationally, <1% of feed is imported (Food and Agriculture Organization of the United Nations, 2017) so it was assumed that all feed was grown in India. We include a more comprehensive description of the land use footprint calculations in Supplementary files 1 and 2.

We include food group-specific footprint values for all indicators in Supplementary Table 2.

### 2.2 Scenario analysis

We measured the change in environmental footprints between current average diets, and two dietary scenarios of shifting to national healthy guidelines, and to affluent diets. We modelled the healthy diets scenario nationally, and for several sub-national samples, including by region (north, north-east, east, south, west, central), rural or urban residence, and for those whose estimated individual-level dietary energy was below (BRI) or above (ARI) recommended age- and sex-specific energy intake. The BRI and ARI groups were meant to represent a simplified picture of the dual challenges of under-nutrition and overweight/obesity, and to highlight broad dietary and environmental changes required to bring these sub-groups to a healthy diet scenario. The affluent diet scenario was assessed for all the same sub-national samples, except for the BRI and ARI groupings. We calculated both relative and absolute changes in environmental footprints, per capita per day.

Dietary guidelines were taken from the Indian National Institute of Nutrition (NIN) (National Institute of Nutrition, 2011), using guidelines on total energy intake (assuming moderate physical activity), % energy from protein and fat (recommended as 10–15 and 20–30%, respectively), and adequate fruit and vegetable intake (excluding intake of potatoes). Dietary energy, fruit and vegetable intake guidelines varied by age and sex (Supplementary Table 3). These guidelines match those of the WHO (Nishida et al., 2004). The age and sex distribution of each of the regional, rural or urban, and BRI/ARI sub-samples was used to create relevant weighted dietary guidelines for each sub-sample.

A healthy diet was optimised for each population sub-sample, with the primary function of minimising deviation from the current diet (the summed and squared relative difference across all food groups) to keep dietary change as realistic as possible (Darmon et al., 2002). Intake of each of the 34 food groups were the variables optimised in the model, and these were also weighted by their relative share of intake in the diet. Our optimisation model minimised the following function:

$${\left(\left(p_{1}\right)\frac{x_{b1}-x_{s1}}{x_{b1}}\right)}^{2}+{\left(\left(p_{2}\right)\frac{x_{b2}-x_{s2}}{x_{b2}}\right)}^{2}+\ldots {\left(\left(p_{n}\right)\frac{x_{\mathrm{bn}}-x_{\mathrm{sn}}}{x_{\mathrm{bn}}}\right)}^{2}$$

where *x* is the intake (grams per day) of food items 1, …,*n*, for optimised healthy (*x*_*s*_) and baseline (*x*_*b*_) diets, and *p* is the proportion of that food item by weight in the diet. We additionally constrained the model to meet the age- and sex-weighted dietary guidelines described above (Supplementary Table 3), and restricted the relative change in intake of any food group to <50%.

Rising incomes are associated with shifts to both greater dietary diversity and excess dietary energy, sugar, and salt intake (Imamura et al., 2015), and we modelled an “affluent diet” scenario to explore how rising incomes may impact diet-related environmental footprints. This scenario assumed the universal adoption of diets that are currently typical of high-income households, which we approximated as the top quartile of households in terms of mean per capita expenditure (MPCE). We generated household MPCE quartiles separately within each of the six Indian regions described above, and by rural or urban residence (twelve total stratifications). Within each of the twelve regional stratifications, individuals from non-affluent households were then assigned the same diets as those from the affluent households, matched for age and sex (e.g. diets of non-affluent individuals from rural central India were shifted to the age- and sex-matched diets of affluent individuals of rural central India). The changes in environmental impacts from this shift were then calculated. We did not conduct a measure of statistical significance, as using the national diet expenditure data results in very small margins of error (while the real uncertainty is likely much larger and a function of measurement error rather than sample size (Tandon and Landes, 2012)), and standard errors were not available in all the environmental footprint data.

Optimisation of healthy diets was modelled using Microsoft Excel's Solver package (specifically using the GRG non-linear algorithm). All other calculations were performed using STATA 13.0.

## 3 Results

### 3.1 Current average diets

Current average intake in India was below recommended guidelines for dietary energy (2141 vs. 2211 kcal/*capita*/day), and fruit and vegetable intake (155 vs. 266 g/*capita*/day, and 83 vs. 98 g/*capita*/day, respectively) (Table 1). The north region was the only exception (comprising the states of Chandigarh, Delhi, Haryana, Himachal Pradesh, Jammu & Kashmir, Punjab, and Uttarakhand), with average intake of dietary energy above recommended levels. Average percentage of dietary energy from protein and fat were adequate nationally, though fell short for fat in some regions. Cereals made up the largest contribution to dietary energy. Contribution from meat was low for all regions (1–3%), while that for dairy varied greatly across regions, ranging from 3% in the north-east to 17% in the north region (Table 1). Compared to national average intake, the BRI population sample had a larger gap between current and recommended consumption of fruit and vegetables, and a higher proportion of dietary energy from cereals. Conversely, compared to national average intake, the ARI sample had greater intake of all 34 food groups assessed, resulting in dietary energy intakes well above recommended guidelines (2534 vs. 2131 kcal/*capita*/day, respectively), with adequate fruit intake, and vegetable consumption greater than the national average but below that recommended by guidelines (Table 2). Mean diet-related environmental footprints nationally per capita per day were 1.3 kgCO_2_-eq, 0.5 m^3^ blue WFs, 1.6 m^3^ green WFs, and 3.9 m^2^ land use (Supplementary Table 4). Food groups which contributed most to diet-related environmental footprints in India were as follows: dairy for GHG emissions, wheat for blue water footprint, rice for green water footprint, and vegetable oils for land use (Supplementary Fig. 1).

**Table 1:** Selected dietary characteristics by Indian regions (per capita).

	North	North east	East	South	West	Central	India
Proportion of population	8%	4%	22%	22%	15%	30%	–
Mean energy intake (kcal)	2337	2064	2139	2093	2091	2158	2141
Dietary guidelines for energy (kcal)^a^	2236	2253	2201	2232	2236	2178	2211
Mean vegetable intake (g)	197	164	170	149	151	137	155
Dietary guidelines for vegetables (g)	269	271	265	270	269	262	266
Mean fruit intake (g)	82	53	44	156	105	52	83
Dietary guidelines for fruit (g)^a^	97	98	98	97	97	98	98
% energy from protein	12%	11%	11%	11%	11%	12%	11%
% energy from fat	25%	13%	15%	21%	27%	19%	20%
% calories from							
Cereals	50%	73%	68%	58%	51%	63%	61%
Pulses	5%	4%	4%	6%	8%	5%	5%
Meat (egg, fish)	1%	3%	2%	3%	1%	1%	1%
Dairy	17%	3%	4%	7%	9%	9%	8%
Fruit and veg	5%	4%	4%	8%	6%	4%	5%
Oils	11%	8%	9%	11%	16%	10%	11%
Other	11%	6%	9%	6%	10%	9%	8%

a As the dietary guidelines are age- and sex-specific, the guideline target is age- and sex-weighted for each region. Targets for dietary energy from protein and fat were recommended as 10–15% and 20–30%, respectively. Regions defined as: North (Chandigarh, Delhi, Haryana, Himachal Pradesh, Jammu & Kashmir, Punjab, Uttarakhand); North-East (Arunachal Pradesh, Assam, Manipur, Meghalaya, Mizoram, Nagaland, Sikkim, Tripura); East (Bihar, Jharkhand, Orissa, West Bengal); South (Andhra Pradesh, Andaman & Nicobar Islands, Karnataka, Kerala, Lakshadweep, Puducherry, Tamil Nadu); West (Dadra & Nagar Haveli, Daman & Diu, Goa, Gujarat, Maharashtra); Central (Chattisgarh, Madhya Pradesh, Rajasthan, Uttar Pradesh). Population proportions sum to 101% due to rounding.

**Table 2:** Selected dietary characteristics of Indian population sub-samples and scenarios used in analysis.

	India	India rural	India urban	BRI	ARI	Affluent
Proportion of Indian population	–	71%	29%	58%	42%	25%
Mean energy (kcal)	2141	2150	2119	1855	2534	2477
Dietary guidelines for energy (kcal)	2211	2197	2244	2269	2131	–
Mean vegetable intake (g)	155	150	165	134	183	191
Dietary guidelines for vegetables (g)	266	265	270	272	258	–
Mean fruit intake (g)	83	63	134	59	116	163
Dietary guidelines for fruit (g)	98	98	98	98	97	–
% energy from protein	11%	11%	11%	11%	11%	11%
% energy from fat	20%	18%	24%	19%	21%	23%
% calories from						
Cereals	61%	64%	53%	63%	59%	53%
Pulses	5%	5%	6%	5%	5%	6%
Meat (egg, fish)	1%	1%	2%	1%	1%	2%
Dairy	8%	7%	10%	7%	10%	12%
Fruit and veg	5%	4%	7%	5%	6%	7%
Oils	11%	10%	13%	11%	11%	11%
Other	8%	8%	9%	8%	9%	9%

Note: BRI, estimated individual-level dietary energy below recommended age- and sex-specific intake; ARI, dietary energy above recommended intake; Affluent, diets of the top quartile of the population according to monthly per capita expenditure. Targets for dietary energy from protein and fat were recommended as 10–15% and 20–30%, respectively. Targets not shown for affluent diet as it was not optimised for health.

### 3.2 Shifts to healthy diets

**Fig. 1:**
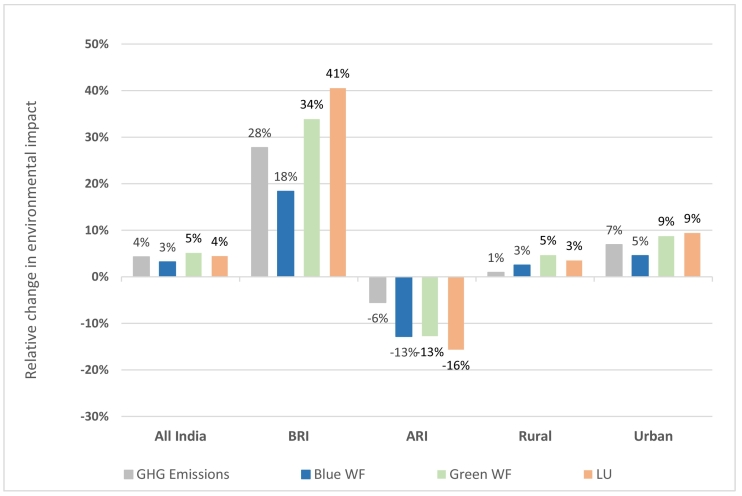
Relative change in greenhouse gas (GHG) emissions, water footprints (WFs), and land use (LU), from shifting current average Indian diets in different population groups to healthy guidelines. Note: BRI, estimated individual-level dietary energy below recommended age- and sex-specific intake; ARI, dietary energy above recommended intake; GHG, greenhouse gas; WF, water footprint; LU, land use. (For interpretation of the references to color in this figure, the reader is referred to the web version of this article.)

Shifts from current average intakes to healthy diets at the national level would result in a small increase of 4% in GHG emissions and LU, and 3 and 5% in blue and green WFs, (and absolute increases of 0.06 kgCO_2_-eq in emissions, 0.02 m^3^ blue WFs, 0.08 m^3^ green WFs, and 0.17 m^2^ LU), respectively (Fig. 1, Supplementary Table 4). The dietary change required to achieve a healthy diet was largely characterised by increased vegetable intake (Supplementary Fig. 2).

However, there were substantial differences in direction of change of environmental footprints among populations below and above recommended dietary energy intake. For those currently below recommended guidelines, the additional agricultural production required to meet healthy guidelines would result in increases of 28% in GHG emissions, 18 and 34% in blue and green WFs, respectively, and 41% in LU (Fig. 1, Supplementary Fig. 3); in absolute terms, equating to increases of 0.31 kgCO_2_-eq emissions, 0.09 m^3^ blue WFs, 0.46 m^3^ green WFs, and 1.39 m^2^ LU (Supplementary Table 4). Meeting dietary guidelines in this sample required increases across a range of food groups (particularly fruit, pulses, vegetables and vegetable oil), while the environmental impacts of this shift were largely driven by meat and vegetables for GHG emissions, vegetable oils and meat for LU, while more distributed across cereals, fruit, meat, vegetables, pulses and vegetables oils for blue and green WFs (Table 2, Table 3).

**Table 3:**
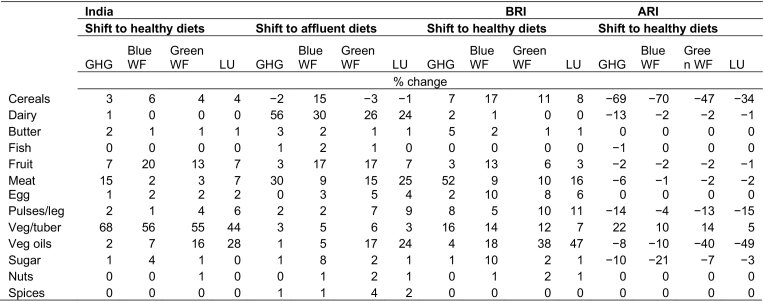
Relative contribution of food groups to changes in greenhouse gas (GHG) emissions, water footprints (WFs) and land use (LU) in the dietary change scenarios.

Note: BRI, estimated individual-level dietary energy below recommended age- and sex-specific intake; ARI, dietary energy above recommended intake; GHG, greenhouse gas; WF, water footprint; LU, land use.

Conversely, for populations above recommended dietary energy intake, decreases of 6% in GHG emissions (−0.09 kgCO_2_-eq), 13% in blue and green WFs (−0.08 and −0.23 m^3^, respectively), and 16% in LU (−0.73 m^2^) could be achieved by meeting healthy guidelines (Fig. 1, Supplementary Table 4). This scenario was largely characterised by lower absolute intake of cereals and sugar in exchange for higher vegetable intake (Supplementary Fig. 4), and the decreases in environmental footprints were mostly due to lower intake of cereals (largely rice), as well as vegetable oils specifically for green WFs and LU (Table 2, Table 3).

### 3.3 Shifts to affluent diets

**Fig. 2:**
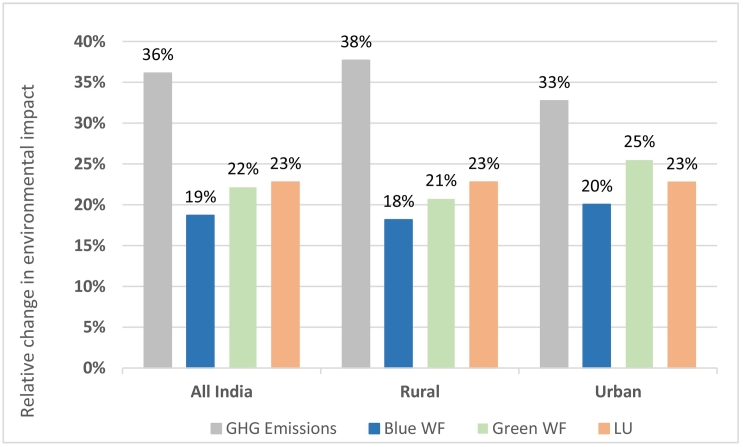
Relative change in greenhouse gas (GHG) emissions, water footprints (WFs), and land use (LU), from shifting current average Indian diets to affluent diets. Note: GHG, greenhouse gas; WF, water footprint; LU, land use. (For interpretation of the references to color in this figure, the reader is referred to the web version of this article.)

We modelled a change to affluent diets to provide a comparative scenario of dietary change based on economic growth, rather than efforts to converge intakes to healthy guidelines. Affluent diets were characterised by high dietary energy (2477 kcal/*capita*/day vs. an age- and sex-weighted recommended dietary energy of 2284 kcal/*capita*/day), high intake of fruit and vegetables (though the latter below guidelines), and compared to average Indian diets, higher dairy and meat intake, and proportion of energy from fats (Table 2). Shifting the entire Indian population to affluent diets would increase GHG emissions by 36% (0.48 kgCO_2_-eq), blue and green WFs by 19 and 22% (0.10 and 0.35 m^3^), respectively, and LU by 23% (0.90 m^2^), with some difference in these changes between rural and urban areas (Fig. 2). Relative to the small increases in environmental footprints required to improve diets nationally in the earlier healthy guidelines scenario, this comparative trajectory to affluent diets would result in substantially higher environmental footprints. This increase in footprints was largely due to higher intake of meat and dairy, while vegetable oils also contributed substantially to the increase in LU and green WFs, and fruit to the increase in blue WFs (Table 3).

### 3.4 Regional analysis

Environmental impacts for both healthy and affluent scenarios varied between the six major Indian sub-regions (Supplementary Table 4). For example, shifts to healthy diets would reduce GHG emissions by 2% in the east, and increase emissions by 7% in the west region. The north would have the largest footprint reductions (−5 and −8% in green WFs and LU, respectively), while the north-east region would experience some of the largest increases (16 and 20% in green WFs and LU respectively). Shifts to affluent diets would increase environmental footprints in all regions. The largest increases would be seen in the central (40% for GHG emissions), south (23% for blue WFs), north (27% for green WFs), and north-east regions (28% for LU).

## 4 Discussion

This study estimates changes in environmental footprints that would result from shifting current national diets to scenarios of healthy or affluent diets, in the context of India's dual burden of under-nutrition and overweight/obesity. Given that dietary shifts could present trade-offs across environmental indicators (Aleksandrowicz et al., 2016), our study extends recent work in LMICs, and is the first to our knowledge to combine nationally-representative Indian dietary data with a range of environmental footprints. Modelling the important goal of adoption of healthy diets for all individuals nationally, we show that increases of about 20–40% across agricultural GHG emissions, blue and green WFs, and LU may be required to shift those currently below recommended dietary energy intake to healthy diets. However, these impacts could be balanced by the opportunity of decreased environmental footprints from healthy shifts among those above recommended dietary energy intake. Overall, only small increases in environmental footprints would result from shifting national-level intakes to diets which are healthy and diverse. Comparatively, a trajectory to affluent diets, typical of the nutrition transition unfolding in LMICs, would lead to additional footprints of 36%, 19%, 22% and 23% in GHG emissions, blue and green WFs, and LU, respectively.

Various food groups contributed to these shifts across the scenarios and sub-populations studied. For example, in a transition to an affluent diet, meat and dairy were largely responsible for the increase in all environmental footprints. A decrease in cereals and oils drove the environmental benefits of shifting the ARI subsample to healthy diets, while a broad diversification of increased intake across pulses, vegetables, and meat drove increases in footprints for the BRI subsample.

Many studies over the last decade have now assessed the potential of using dietary change to improve health and environmental outcomes, though this literature has almost exclusively been focused on HICs, and analyses at the global level have not specifically assessed the impacts of improving diets for potentially undernourished populations (Aleksandrowicz et al., 2016). Recent work has begun to examine these relationships in LMICs (Milner et al., 2017; Rao et al., 2018; Arrieta and Gonzalez, 2018; Bahn et al., 2018; Lei and Shimokawa, 2017; Song et al., 2017). In China, two recent analyses found that national shifts to healthy diets would decrease footprints; in one case, annual national GHG emissions and blue WFs reduced by 1.7 ∗ 10^12^ g and 2.7 ∗ 10^13^ L, respectively (comparatively, using the 2012 Indian population (The World Bank, 2019), our results indicated an annual national increase of 2.8 ∗ 10^13^ g and 9.2 ∗ 10^12^ L, respectively) (Lei and Shimokawa, 2017), and a second analysis showed GHG emissions decreasing by about 12% (Song et al., 2017). These results are contrary to our analysis for India, though can likely be explained by China's lower rates of undernutrition (International Food and Policy Research Institute, 2017a; International Food and Policy Research Institute, 2017b), and a higher baseline intake of meat than in India, the reduction of which contributed to much of the environmental benefit of healthy diets. A study at the city level in Delhi assessed improving nutrition status for the poorest half of the population to that of the median income class, and found modest increases – 4–9% across the same three environmental indicators (Boyer and Ramaswami, 2017) – lower than those found here, as the dietary energy gap existing in their scenario was smaller than the one we examined. Milner et al. (2017) found that across several Indian regions, shifts to healthy diets could be protective against future water-related pressures facing agricultural production, and additionally decrease GHG emissions. The most comparable study to ours, also using national data, concluded that meeting micronutrient requirements could reduce GHG emissions by 19% (Rao et al., 2018) (though a scenario of minimising deviation from baseline diets saw a smaller reduction); this contrasts with our results, which saw a small increase of 4%. This could be a function of several differences between our analyses, including underlying GHG values (the authors' animal-based food footprint values were greater than ours), a healthy diet definition focused on micronutrients compared to our use of absolute fruit and vegetable intake, and use of the ‘type 1’ NSS format compared to our use of ‘type 2’ (30-day vs. 7-day recall). Our healthy scenario optimisation minimised deviation from current intakes to model a realistic dietary shift; this healthy scenario was marked by little change in intake of cereals, with substantial increase in vegetable intake, and to a lesser extent, fruit. The analysis by Rao et al. highlighted that within cereals, shifts from fine rice to wheat and other coarse grains, could be another important route for health and environmental benefits (Rao et al., 2018).

We have highlighted dietary change among those who consume adequate dietary energy as a pathway to reducing environmental footprints. However, given the importance of improving nutrition for all within current environmental pressures, this demand-side approach should be viewed as only one pathway alongside others (Lamb et al., 2016; Bryngelsson et al., 2016; Erb et al., 2016). For example, supply-side measures could offer substantial environmental benefits in India, such as tackling food loss (Balaji and Arshinder, 2016; Hic et al., 2016), closing yield gaps (Carlson et al., 2017; Pradhan et al., 2015) improving efficiency of livestock production (Herrero et al., 2013; Food and Agriculture Organization, 2013), and wider adoption of multiple cropping. Much of the sustainable diets literatures focuses on HICs and associated GHG emissions of dietary change, though for some LMICs such as India, water and land use pressures may be particularly urgent (Weinzettel et al., 2013; Kastner et al., 2012; Alexander et al., 2015). For example, cultivatable land in India has decreased in recent decades, and with competing demands for land, there is little room to increase agricultural land area (Ministry of Agricultre and Farmers Welfare, 2016). Given that achieving healthy diets for undernourished individuals would result in additional pressures on agricultural production, the importance of these other agricultural improvements is of high priority – and implementing them could more than offset the environmental pressures of providing healthy diets nationally. The urgency of implementing these solutions will also likely increase in the near term, as environmental change is projected to exacerbate dietary and environmental challenges by lowering yields and nutritional quality of crops (Loladze, 2014; Scheelbeek et al., 2018; Smith and Myers, 2018).

The lesson from most countries globally is that economic development does not necessarily result in consumption of a healthy diet (Global Panel on Agriculture and Food Systems for Nutrition, 2016b; Imamura et al., 2015); rising incomes may shift people from undernutrition, but introduce NCD risks, such as those due to excess dietary energy, sugar, oils, and salt. We have shown that a simplified scenario of this trajectory in India would also result in substantially increased environmental footprints. This trajectory may not be inevitable, as the example of South Korea shows, where incomes have grown rapidly while obesity and other NCDs have remained relatively low (Global Panel on Agriculture and Food Systems for Nutrition, 2016b). Navigating the dual burden of malnutrition will, however, require marked efforts to implement a comprehensive and coordinated suite of policies across the food system, for example, in improved production, distribution and storage of nutrient-rich crops, subsidies and taxes for relevant foods, education on healthy diets, and regulating advertising and content of processed food.

Our study has several limitations. The analysis uses hypothetical scenarios, and should be interpreted as indicative of broad opportunities and challenges, rather than as projections. The shifts to various scenarios did not include other potential drivers such as trade or environmental pressures on food production that may affect the availability or affordability of food, and therefore the makeup of the dietary scenarios. Similarly, we were unable to model dynamically how dietary environmental footprints may fluctuate in response to changing intakes and associated agricultural production. We used average national values of environmental footprints. While the analysis could be improved with the use of more granular footprint data, where available, incorporating these would require more detailed knowledge than is currently available on the regional source of food groups consumed in any given state. We also did not include future projections of population, though this more clearly highlights the challenges of addressing the dietary gaps between current intakes and healthy diets. The underlying environmental data used proxies for some food items, as detailed environmental data for all foods eaten are not available, and are rare even for HICs. However, much of the literature attributing environmental impacts to diets uses similar methodology, and previous work has shown that using simplified food groups as proxies is a valid approach (Pernollet et al., 2017; Heller et al., 2018). We had also assumed that all food in our dietary scenarios is domestically produced (this is true for the majority of food consumed in India (Food and Agriculture Organization of the United Nations, 2017)), and using this approach allowed us to gauge the total environmental impacts, though future analyses can be improved by combining international and intra-national trade data. The NSS dietary data recorded the expenditure on meals outside of the home, from which we estimated food group intake. However, the food groups eaten outside the home may be different from those which are recorded as purchased for the household, and the out-of-home data may underestimate total intake (Aleksandrowicz et al., 2017). Micronutrient deficiencies remain a substantial challenge in India (National Nutrition Monitoring Bureau, 2012), though adherence to micronutrient RDAs in the optimised healthy diet would be difficult to reliably assess with the use of household expenditure data. We used the high-level, public-facing recommendations from the NIN (National Institute of Nutrition, 2011) on macronutrients (energy, protein, and fats), and adequate fruit and vegetable intake, which match those of WHO/FAO (Nishida et al., 2004). We have assumed that meeting these intakes would provide a realistic and transparent healthy diet scenario, though our modelling may have assigned some individual-level intakes as healthy without fully aligning with additional micronutrient requirements. Affordability is an important consideration in the feasibility of shifting to healthy diets. We have not assessed the cost of our dietary shifts, though as the dietary guidelines we model are based on existing recommendations, we focused on the environmental impacts of achieving these public health goals. The expenditure data itself may not represent actual intakes, and substantial variation in dietary intakes was found in a comparison of dietary datasets in India, with particular discrepancies for nutrient-dense foods such as fruit and animal-based products (Aleksandrowicz et al., 2017). However, under- or over-estimates of intake may have, to some extent, been cancelled out in the affluent diet scenarios, as for example, both baseline and scenario diets would likely include the same direction of measurement error. Also, we were not able to provide measures of error across the environmental footprints, as these are unavailable for the LU data, and inputs to generate uncertainty are not consistent across the WF and GHG data, which would produce incomparable uncertainty ranges. We are not aware of any other studies that have generated uncertainty ranges across several environmental indicators, and the methodology in this area remains a topic for further work. Additionally, the artificially narrow confidence intervals in the large national dietary data would not accurately represent true uncertainty ranges of intake. One of the strengths of our study is using a variety of environmental indicators, though a more comprehensive assessment could include additional outcomes for which data were not available to us, such as biodiversity and nutrient flows.

Future work is necessary to add finer detail to the environmental data, and understand implications at smaller spatial scales by, for example, using sub-national data on trade, production location, and environmental impacts with greater resolution, as pressures such as water stress vary considerably by region. In addition, interdisciplinary research will be vital to better understand the complex linkages between environment, food, and health (Picchioni et al., 2017).

India suffers a dual burden of malnutrition. Widespread adoption of healthy diets could generate substantial public health benefits through reducing hunger and nutrient deficiencies, and reducing risks of diet-related NCDs such as diabetes and hypertension. However, unlike in HICs, our study has demonstrated that widespread adoption of healthy diets may not reduce environmental footprints of the Indian food system relative to the status quo, albeit preferable to the widespread adoption of diets currently consumed by the wealthiest quartile of the population. Thus, to achieve improved population health and reduced environmental impacts, additional strategies to reduce food waste and increase the efficiency of food production will be required.

## Funding

LA's studentship is funded through the Leverhulme Centre for Integrative Research on Agriculture and Health. This study contributes to the Sustainable and Healthy Diets in India (SADHI) and the Sustainable and Healthy Food Systems (SHEFS) programmes supported by the Wellcome Trust's Our Planet, Our Health programme (grant numbers: 103932/Z/14/Z and 205200/Z/16/Z). The funders of this study had no role in study design, data collection, data analysis, data interpretation, or writing of the report.

## Appendix A Supplementary data

Supplementary materials: figures and tables. Image 1

Supplementary File 1 Details on dietary adjustments and land use values. Supplementary File 1

Supplementary File 2 Land use footprint calculations. Supplementary File 2
