# Supplementary material for: Environmental impacts of dietary shifts in India: A modelling study using nationally-representative data
Source: Environ Int. 2019 May;126:207–15. doi: 10.1016/j.envint.2019.02.004 (PMC6437131; doi:10.1016/j.envint.2019.02.004)
Supplement: Supplementary file 1 — Supplementary materials: figures and tables. [file mmc1.pdf]

Supplementary figure 1: Contribution of food groups to total dietary greenhouse gas (GHG) emissions, blue and green water footprints (WFs), and land use (LU)

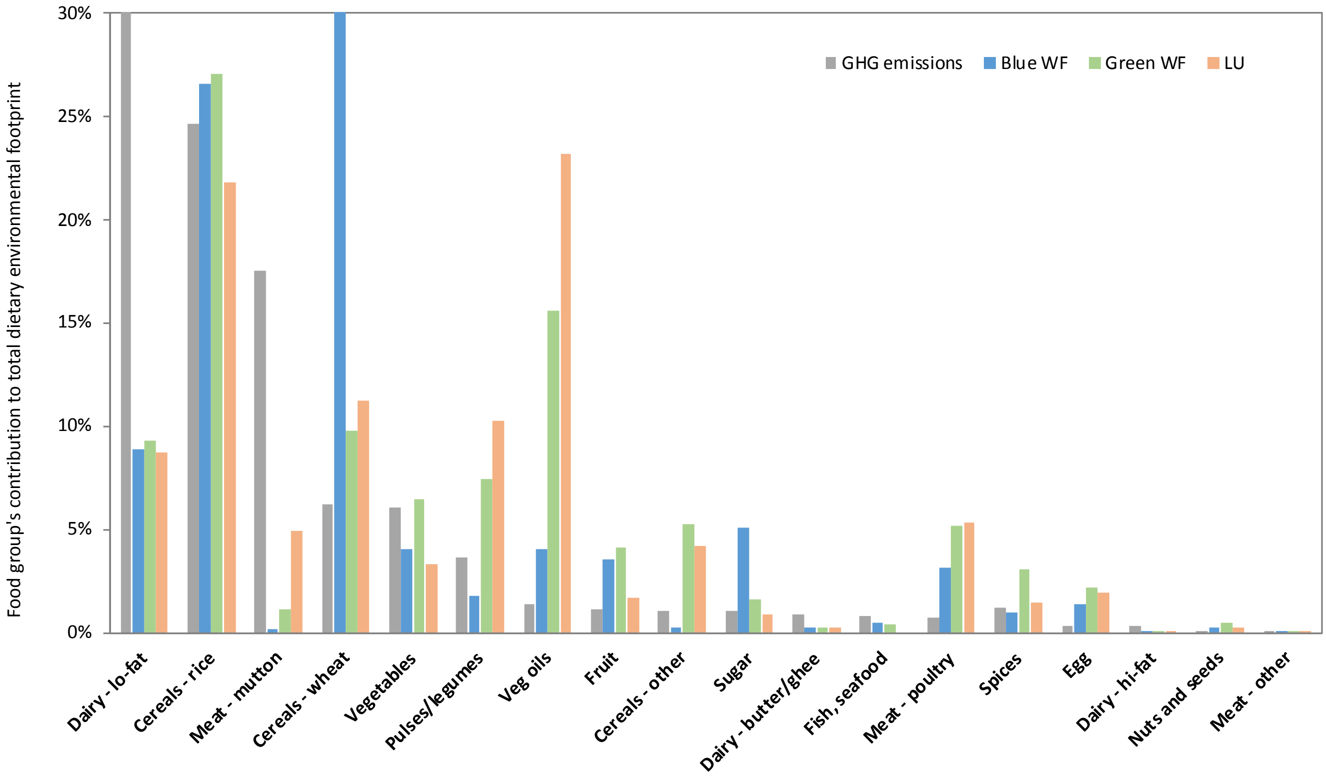

Note: GHG emissions from the dairy lo-fat category are 32%, and blue WF of the wheat category is 39%.

Supplementary figure 2: Intake of food groups in current average diets and optimised healthy diets, nationally

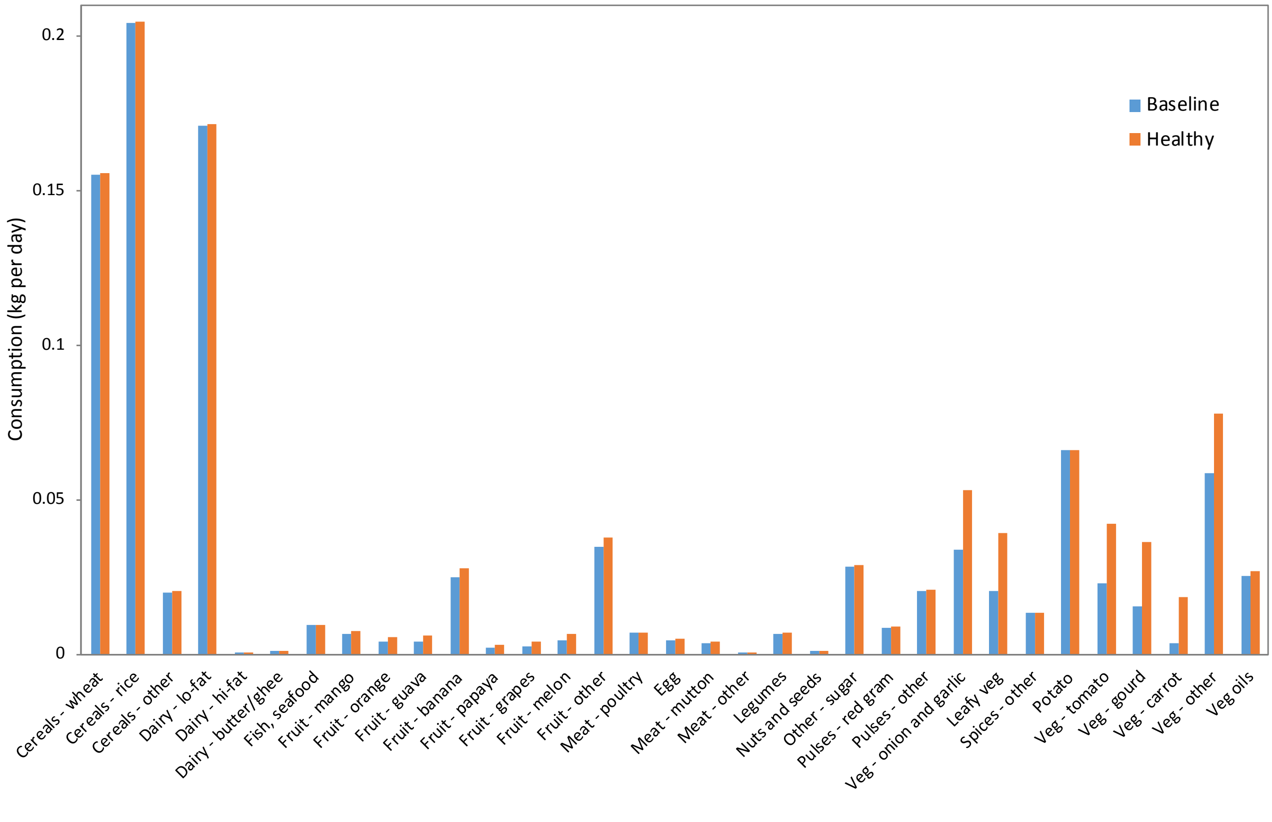

Supplementary figure 3: Intake of food groups in current average diets and optimised healthy diets among those estimated to be below recommended dietary energy intake, nationally

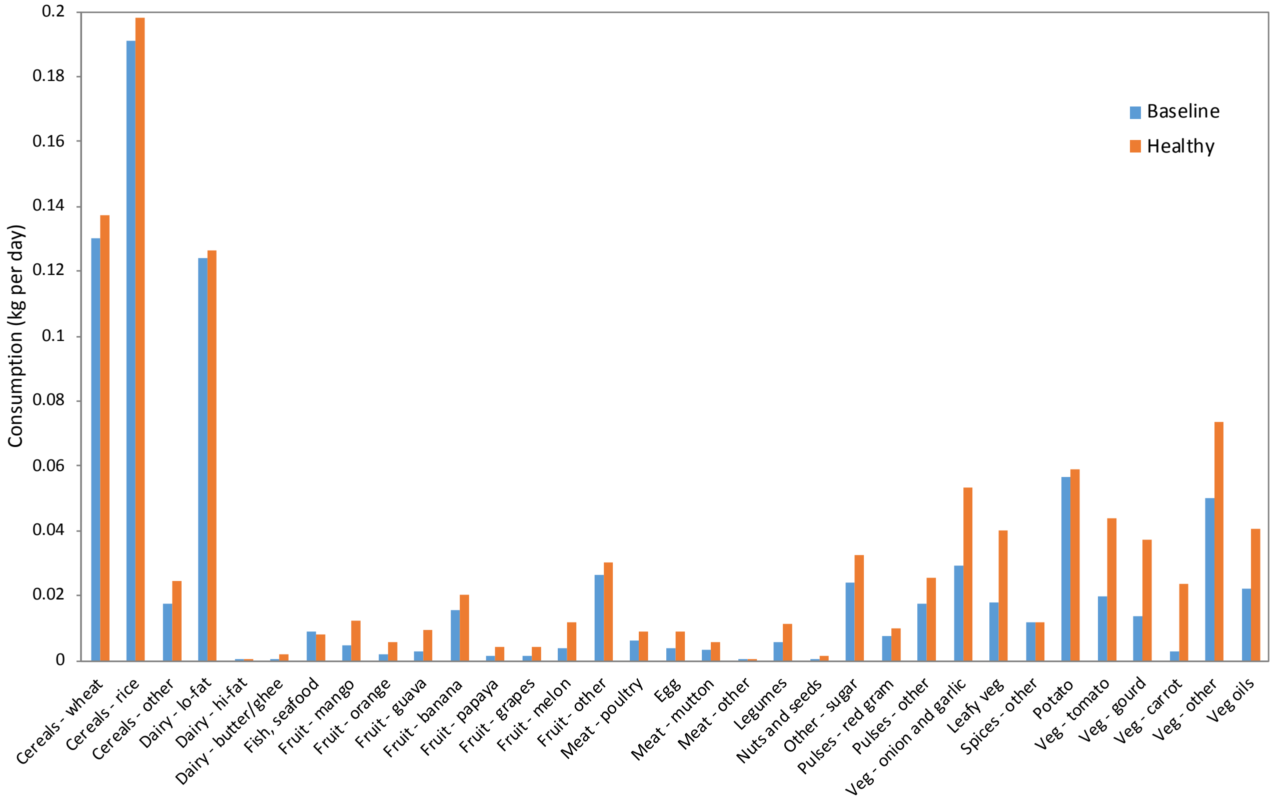

Supplementary figure 4: Intake of food groups in current average diets and optimised healthy diets among those estimated to be above recommended dietary energy intake, nationally

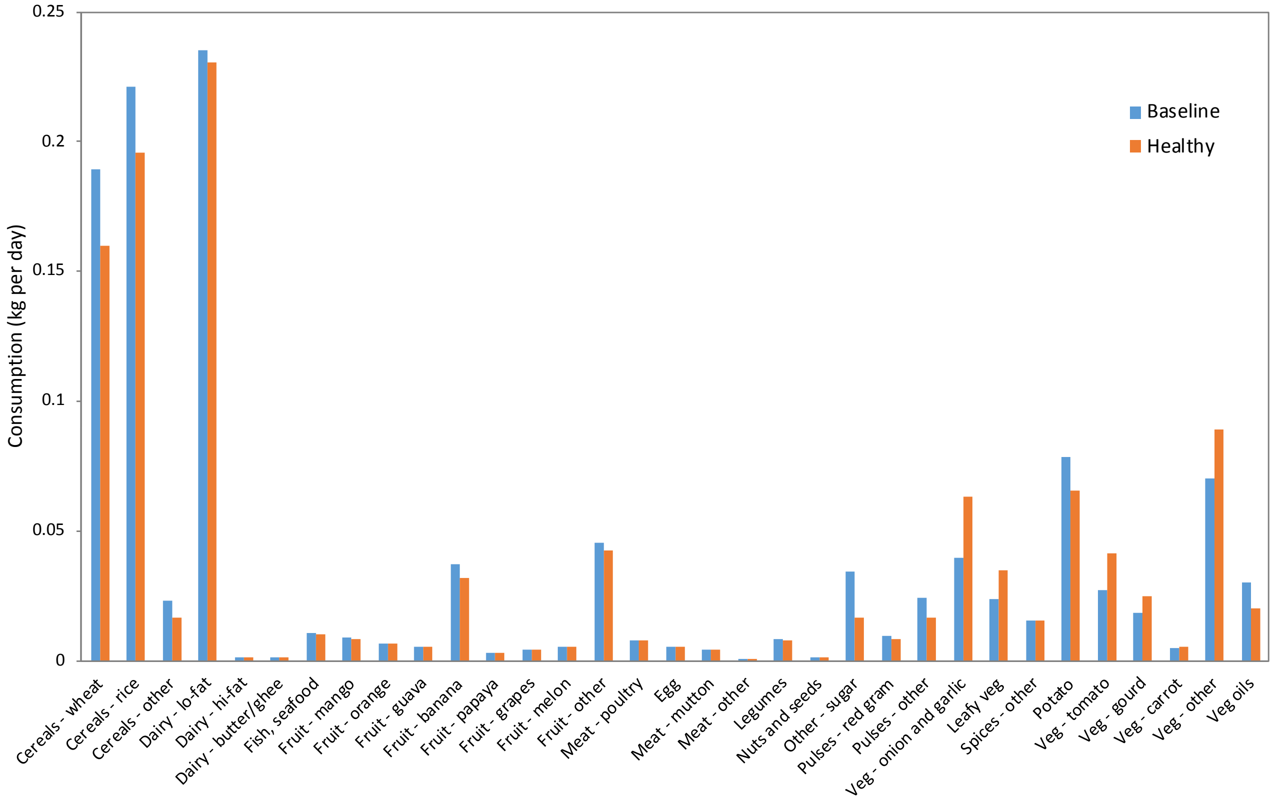

Supplementary table 1: Allocation of individual food items to food groups

Food group	Original food item
Cereals - wheat	wheat/atta
	maida
	suji, rawa
	sewai, noodles
	bread bakery
	other wheat products
Cereals - rice	rice
	chira
	khoi, lawa
	muri
	other rice products
Cereals - other	jowar & products
	bajra & products
	maize & products
	barley & products
	small millets &products
	ragi & products
	other cereals
Dairy - lo-fat	milk liquid
	baby food
	milk condensed/ powder
	curd
Dairy - hi-fat	ice-cream
	other milk products
Dairy - butter/ghee	ghee
	butter
Fish, seafood	fish, prawn
Fruit - mango	mango
Fruit - orange	orange, mausami
Fruit - guava	guava
Fruit - banana	banana
Fruit - papaya	papaya
Fruit - grapes	grapes
Fruit - melon	watermelon
	kharbooza
Fruit - other	jackfruit
	pineapple
	coconut
	green coconut
	singara
	pears/nashpati
	berries
	leechi
	apple
	other fresh fruits
	lemon
	dates
	raisin, kishmish, monacca,etc.
	coconut copra
	other dry fruits
Meat - poultry	chicken

Egg	eggs
Meat - mutton	goat meat/mutton
	beef/ buffalo meat
Meat - other	pork
	others birds, crab,oyster, tortoise, etc.
Legumes	beans, barbati
	groundnut
Nuts and seeds	cashewnut
	walnut
	other nuts
Sugar	sugar
	gur
	candy, misri
	honey
Pulses - red gram	arhar, tur
Pulses - other	gram split
	gram whole
	moong
	masur
	urd
	peas
	khesari
	other pulses
	gram products
	besan
	other pulse products
Veg - onion and garlic	onion
	garlic
Leafy veg	palak/other leafy vegetables
Spices - other	ginger
	jeera
	dhania
	turmeric
	black pepper
	dry chillies
	tamarind
	curry powder
	oilseeds
	other spices
Potato	potato
Veg - tomato	tomato
Veg - gourd	parwal/patal, kundru
	gourd, pumpkin
Veg - carrot	carrot
Veg - other	brinjal
	radish
	green chillies
	lady's finger
	cauliflower
	cabbage
	peas
	other vegetables

Veg oils	edible vegetable oils (mustard, groundnut, coconut, sunflower, soyabean, saffola, others)
	vanaspati, margarine

Supplementary table 2: Environmental footprints of food groups

Food group	GHG emissions ($kgCO_2$-eq/kg food)	Green WF (m^3/kg food)	Blue WF (m^3/kg food)	Land use (m^2/kg food)
Cereals - wheat	0.540	0.985	1.366	2.867
Cereals - rice	1.614	2.072	0.715	4.227
Cereals - other	0.725	4.171	0.066	8.468
Dairy - lo-fat	2.524	0.845	0.285	2.016
Dairy - hi-fat	6.851	2.718	0.917	4.732
Dairy - butter/ghee	12.531	4.321	1.458	10.268
Fish, seafood	1.172	0.670	0.295	-
Fruit - mango	0.115	1.237	0.566	1.517
Fruit - orange	0.264	0.678	0.003	1.010
Fruit - guava	0.117	1.237	0.566	1.606
Fruit - banana	0.195	0.266	0.193	0.338
Fruit - papaya	0.117	0.267	0.087	0.278
Fruit - grapes	0.586	0.289	0.336	0.510
Fruit - melon	0.610	0.265	0.025	0.559
Fruit - other	0.117	1.158	0.218	1.038
Meat - poultry	1.425	11.782	2.530	30.631
Egg	1.119	7.721	1.658	16.987
Meat - mutton	63.531	4.872	0.290	52.913
Meat - other	1.425	3.324	0.272	5.162
Legumes	1.759	1.606	0.129	4.869
Nuts and seeds	1.286	9.410	1.525	13.358
Other - sugar	0.504	0.925	0.995	1.294
Pulses - red gram	1.398	5.068	0.282	18.213
Pulses - other	1.261	3.114	0.323	10.814
Veg - onion and garlic	0.740	0.164	0.143	0.653
Leafy veg	0.155	0.412	0.046	0.466
Spices - other	1.254	3.626	0.424	4.521
Potato	0.497	0.218	0.038	0.565
Veg - tomato	0.138	0.223	0.043	0.523
Veg - gourd	0.212	0.375	0.012	1.301
Veg - carrot	0.703	0.082	0.060	0.724
Veg - other	0.201	1.059	0.217	0.466
Veg oils	0.746	9.541	0.867	35.868

Supplementary table 3: Daily energy, vegetable and fruit intake, according to national dietary guidelines[42]

Sex	Age (years)	Energy (kcal/*capita*/day)	Vegetable intake (g/*capita*/day)	Fruit intake (g/*capita*/day)
Male	<1	584	55	55
	1-3	1060	100	100
	4-6	1350	150	100
	7-9	1690	200	100
	10-12	2190	300	100
	13-15	2750	300	100
	16-17	3020	300	100
	18-59	2730	300	100
	60-69	2184	240	80
	70+	1911	210	70
Female	<1	584	55	55
	1-3	1060	100	100
	4-6	1350	150	100
	7-9	1690	200	100
	10-12	2010	300	100
	13-15	2330	300	100
	16-17	2440	300	100
	18-59	2230	300	100
	60-69	1625	240	80
	70+	1593	210	70

Supplementary table 4: Relative changes in greenhouse gas (GHG) emissions, water footprints (WFs) and land use (LU) from shifting average Indian diets to healthy guidelines and affluent dietary scenarios

	GHG emissions					Blue WF					Green WF					LU				
**Region**	**Baseline**	**Healthy**		**Affluent**		**Baseline**	**Healthy**		**Affluent**		**Baseline**	**Healthy**		**Affluent**		**Baseline**	**Healthy**		**Affluent**	
	**$kgCO_2$-eq**	**$kgCO_2$-eq**	**% diff.**	**$kgCO_2$-eq**	**% diff.**	**m^3**	**m^3**	**% diff.**	**m^3**	**% diff.**	**m^3**	**m^3**	**% diff.**	**m^3**	**% diff.**	**m^2**	**m^2**	**% diff.**	**m^2**	**% diff.**
North rural ARI	2.1	1.95	-6.7			0.8	0.7	-13.4			1.7	1.5	-16.1			4.5	3.6	-20.0		
North rural BRI	1.3	1.5	12.2			0.6	0.6	11.6			1.2	1.6	26.7			3.3	4.2	28.2		
North rural	1.8	1.8	-0.7	2.5	38.5	0.7	0.7	-3.1	0.8	18.1	1.5	1.4	-8.0	1.9	22.5	4.0	3.5	-13.5	4.8	19.8
North urban ARI	2.0	1.9	-5.0			0.8	0.7	-11.4			1.9	1.6	-16.2			4.9	3.9	-20.0		
North urban BRI	1.2	1.4	16.1			0.5	0.6	14.6			1.3	1.6	28.7			3.3	4.4	31.8		
North urban	1.6	1.6	1.6	2.2	40.1	0.6	0.6	1.0	0.8	23.2	1.5	1.6	1.4	2.1	34.5	4.0	4.0	0.7	5.2	29.3
**North total**	**1.7**	**1.7**	**-1.5**	**2.4**	**39.1**	**0.7**	**0.7**	**-1.4**	**0.8**	**20.0**	**1.5**	**1.5**	**-4.9**	**2.0**	**27.2**	**4.0**	**3.7**	**-7.9**	**5.0**	**23.5**
North-east rural ARI	1.6	1.4	-12.6			0.6	0.5	-15.7			2.0	1.8	-6.3			4.6	4.4	-3.2		
North-east rural BRI	1.2	1.4	16.1			0.4	0.5	20.2			1.4	1.8	31.6			3.3	4.6	39.6		
North-east rural	1.4	1.4	-0.3	1.8	29.4	0.5	0.5	5.4	0.5	18.9	1.6	1.8	15.6	2.0	24.5	3.7	4.5	19.7	4.7	27.5
North-east urban ARI	1.7	1.6	-8.1			0.6	0.5	-14.0			2.1	1.9	-8.1			5.0	4.5	-8.6		
North-east urban BRI	1.2	1.5	28.7			0.4	0.5	25.5			1.4	2.0	39.6			3.4	5.1	49.6		
North-east urban	1.3	1.5	13.0	1.8	34.3	0.5	0.5	9.7	0.6	24.6	1.6	1.9	15.5	2.1	27.9	3.9	4.6	19.5	5.1	31.2
**North east total**	**1.4**	**1.4**	**3.1**	**1.8**	**30.1**	**0.5**	**0.5**	**6.4**	**0.5**	**19.7**	**1.6**	**1.8**	**15.8**	**2.0**	**25.0**	**3.7**	**4.5**	**20.3**	**4.8**	**28.0**
East rural ARI	1.5	1.3	-11.3			0.6	0.5	-14.5			1.7	1.7	-3.5			4.2	4.3	1.8		
East rural BRI	1.0	1.1	11.6			0.5	0.5	17.7			1.3	1.7	30.4			3.1	4.2	35.7		
East rural	1.2	1.2	1.3	1.6	35.0	0.5	0.5	-1.5	0.6	14.9	1.5	1.6	11.2	1.8	20.6	3.6	4.1	13.9	4.5	25.7
East urban ARI	1.6	1.5	-5.8			0.6	0.6	-11.8			1.9	1.7	-10.6			4.8	4.2	-12.5		
East urban BRI	1.3	1.7	34.2			0.5	0.6	20.9			1.4	1.9	38.7			3.5	5.2	48.3		
East urban	1.4	1.5	9.6	1.7	23.3	0.5	0.6	6.7	0.6	18.5	1.5	1.7	11.9	2.0	27.3	4.0	4.5	14.3	5.0	25.4
**East total**	**1.2**	**1.2**	**-2.3**	**1.7**	**32.6**	**0.5**	**0.5**	**1.3**	**0.6**	**15.6**	**1.5**	**1.6**	**7.6**	**1.8**	**21.9**	**3.7**	**4.0**	**8.0**	**4.6**	**25.6**
South rural ARI	1.8	1.7	-5.5			0.6	0.5	-12.0			2.3	2.0	-12.7			5.6	4.7	-15.5		
South rural BRI	1.2	1.4	18.4			0.4	0.5	19.6			1.6	2.1	31.0			3.9	5.4	39.6		
South rural	1.4	1.6	8.1	2.0	37.8	0.5	0.5	6.0	0.6	21.9	1.9	2.0	8.0	2.2	19.8	4.5	4.9	9.1	5.5	22.1
South urban ARI	2.0	2.0	-3.9			0.6	0.6	-11.4			2.4	2.0	-12.9			5.7	4.8	-16.3		
South urban BRI	1.3	1.6	18.3			0.4	0.5	19.2			1.6	2.1	30.3			4.0	5.5	38.2		
South urban	1.6	1.7	6.1	2.2	37.9	0.5	0.5	7.0	0.6	25.2	1.9	2.1	9.4	2.3	23.4	4.6	5.1	9.2	5.7	22.5
**South total**	**1.5**	**1.6**	**6.4**	**2.1**	**37.8**	**0.5**	**0.5**	**5.8**	**0.6**	**23.2**	**1.9**	**2.0**	**8.2**	**2.3**	**21.1**	**4.5**	**5.0**	**9.5**	**5.6**	**22.2**
West rural ARI	1.3	1.3	-2.6			0.6	0.5	-8.1			2.0	1.7	-13.9			5.1	4.1	-19.3		

West rural BRI	1.0	1.1	18.2			0.4	0.5	18.8			1.5	1.9	31.0			3.8	5.1	34.6		
West rural	1.1	1.2	7.2	1.5	32.8	0.5	0.5	7.2	0.6	19.7	1.7	1.8	10.2	1.9	16.6	4.3	4.7	9.5	5.0	17.7
West urban ARI	1.8	1.8	-3.0			0.7	0.6	-8.4			2.1	1.8	-14.0			5.4	4.4	-19.0		
West urban BRI	1.2	1.7	43.2			0.5	0.6	21.0			1.5	2.0	33.7			3.9	5.4	36.5		
West urban	1.4	1.6	8.1	1.8	28.5	0.5	0.6	5.0	0.6	16.8	1.7	1.8	9.1	2.0	20.8	4.5	4.9	9.5	5.3	17.9
**West total**	1.2	1.3	6.9	1.6	30.6	0.5	0.5	5.5	0.6	18.4	1.7	1.8	9.7	2.0	18.5	4.4	4.8	9.9	5.1	17.8
Central rural ARI	1.4	1.3	-7.5			0.7	0.6	-12.4			1.6	1.4	-12.9			4.0	3.4	-15.8		
Central rural BRI	0.9	1.1	13.9			0.5	0.6	14.2			1.1	1.5	33.3			2.9	4.0	35.3		
Central rural	1.2	1.1	-2.1	1.7	42.5	0.6	0.6	0.9	0.7	18.1	1.3	1.3	1.7	1.6	22.4	3.5	3.4	-1.5	4.3	23.4
Central urban ARI	1.8	1.7	-4.2			0.7	0.7	-9.9			1.7	1.4	-14.5			4.5	3.7	-18.9		
Central urban BRI	1.2	1.5	31.4			0.5	0.6	16.1			1.1	1.6	36.4			3.2	4.4	38.1		
Central urban	1.4	1.5	6.2	1.9	31.7	0.6	0.6	3.7	0.7	16.8	1.4	1.5	7.5	1.7	27.8	3.8	4.0	6.7	4.6	23.0
**Central total**	1.2	1.2	-0.2	1.7	39.7	0.6	0.6	1.9	0.7	17.8	1.3	1.4	2.7	1.7	23.6	3.5	3.5	-0.3	4.3	23.3
India rural ARI	1.6	1.5	-6.5			0.6	0.6	-13.8			1.8	1.6	-11.4			4.5	3.9	-13.9		
India rural BRI	1.0	1.4	31.4			0.5	0.6	18.7			1.3	1.8	34.5			3.3	4.7	41.3		
**India rural**	1.3	1.3	0.9	1.8	37.7	0.5	0.6	2.5	0.6	18.2	1.5	1.6	4.7	1.8	20.7	3.8	4.0	3.5	4.7	22.8
India urban ARI	1.9	1.8	-4.3			0.7	0.6	-10.4			2.0	1.8	-12.4			5.1	4.2	-17.7		
India urban BRI	1.2	1.5	22.9			0.5	0.6	17.0			1.4	1.9	33.3			3.7	5.1	39.5		
**India urban**	1.5	1.6	6.9	2.0	32.7	0.6	0.6	4.5	0.7	20.0	1.6	1.8	8.7	2.0	25.5	4.2	4.6	9.4	5.2	22.8
**India ARI**	1.6	1.6	-5.5			0.7	0.6	-12.8			1.8	1.6	-12.8			4.7	3.9	-15.6		
**India BRI**	1.1	1.4	27.8			0.5	0.6	18.4			1.4	1.8	33.9			3.4	4.8	40.5		
**India Total**	1.3	1.4	4.3	1.8	36.1	0.5	0.6	3.2	0.7	18.7	1.6	1.6	5.1	1.9	22.1	3.9	4.1	4.4	4.8	22.8
