# Supplementary material for: Environmental impacts of dietary shifts in India: A modelling study using nationally-representative data
Source: Environ Int. 2019 May;126:207–15. doi: 10.1016/j.envint.2019.02.004 (PMC6437131; doi:10.1016/j.envint.2019.02.004)
Supplement: Supplementary File 1 — Details on dietary adjustments and land use values. [file mmc2.docx]

**Supplementary File 1**

***Adjustments made to reported food purchase in National Sample Survey household consumer expenditure data***

Dietary data were adjusted for high-income households that provide food to poorer households in exchange for labour or services as follows:

$$adjusted intake\boldsymbol{=} C_{i}\left( \frac{M_{h}+M_{f}}{M_{h}+M_{g}} \right)$$

where C is the unadjusted intake of food item i, M_h_ is the number of meals consumed by the household members, M_f_ is the number of meals received free from other households by household members, and M_g_ is the number of meals consumer by non-members (guests, employees, etc.).

Data were additionally adjusted for foods eaten out of home. NSS records purchase of ~140 individual foods which we matched to nutritional composition data for our analysis. However, some of the recorded purchases are of a variety meals and snacks outside of home, which we were not able to break down into food groups required for our analysis, such as fruits and vegetables. To approximate intake of food groups from these meals and snacks, we used the NSS data on estimated caloric content of out of home meals. We took the proportion of these out of home calories out of all calories, and then scaled the individual food items purchase by this proportion. This adjustment assumed that the distribution of food groups in the meals purchased out of home was the same as the purchased food items.

***Calculation of land use footprints***

National-level yield data for crops were obtained from the FAO, available for the years 1961–2014 (Table 1; FAOSTAT, 2017). These data provide an estimate of the quantity of individual crop items produced per hectare and are principally derived from national agricultural surveys. Standard technical conversion factors (FAO, 1972) were applied to account for non-edible components (e.g. fruit skin; Table 1). For livestock products, FAOSTAT publish data on yields per head of livestock but they do not publish yields per unit area of land. Thus, yield data for livestock products were calculated as follows: (i) the make-up of feed (i.e. concentrates, grass and non-grass roughages) in grazing, mixed and industrial systems for different animals was derived from Mekonnen and Hoekstra (2012) and Harris et al. (2017; Table 2); (ii) the yield of concentrates was based on FAOSTAT yield and feed production data for cereals, oil crops, and pulses (FAOSTAT, 2017); (iii) the yield of grass was assumed to be 4 kg ha^-1^ year^-1^ (Shankar & Gupta, 1992) and it was considered that production of roughage other than grass (i.e. by-products of other crops) do not require additional land; (iv) the land area required *per* kg of livestock product was calculated on the basis of feed yields and feed conversion efficiencies. This value was inverted to give kg of food product *per* ha of land. Nationally, <1% of feed is imported (FAOSTAT 2017) so it was assumed that all feed was grown in India. Land requirements of fish were not considered.

**References**

Food and Agriculture Organization, FAO. Technical Conversion Factors for Agricultural Commodities. 1972. Available online: <http://www.fao.org/fileadmin/templates/ess/documents/methodology/tcf.pdf> [accessed February 2017].

Food and Agriculture Organization Corporate Statistical Database, FAOSTAT. Food and Agriculture Organization of the United Nations. 2017. Available online: <http://faostat3.fao.org/home/E> [accessed February 2017].

Harris, F., Green, R.F., Joy, E.J.M., Kayatz, B., Haines, A., Dangour, A.D., 2017. The water use of Indian diets and socio-demographic factors related to dietary blue water footprint. Sci. Total Environ. 1, 128-136.

Mekonnen, M.M., Hoekstra, A.Y., 2012. A Global Assessment of the Water Footprint of Farm Animal Products. Ecosystems 15, 401–415.

Shankar, V., Gupta, J.N., 1992. Restoration of Degraded Rangelands. In: J. S. Singh (ed.). Restoration of Degraded Lands-Concepts and Strategies. Rastogi Publications, Meerut, India.Food and Agriculture Organization Corporate Statistical Database, FAOSTAT. Food and Agriculture Organization of the United Nations. 2017. Available online: http://faostat3.fao.org/home/E [accessed February 2017].

Harris, F., Green, R.F., Joy, E.J.M., Kayatz, B., Haines, A., Dangour, A.D., 2017. The water use of Indian diets and socio-demographic factors related to dietary blue water footprint. Sci. Total Environ. 1, 128-136.

Mekonnen, M.M., Hoekstra, A.Y., 2012. A Global Assessment of the Water Footprint of Farm Animal Products. Ecosystems 15, 401–415.

Shankar, V., Gupta, J.N., 1992. Restoration of Degraded Rangelands. In: J. S. Singh (ed.). Restoration of Degraded Lands-Concepts and Strategies. Rastogi Publications, Meerut, India.
